# Development and initial validation of a new self-report measure to assess perceived dependence on tobacco and nicotine products

**DOI:** 10.1038/s41598-024-60790-4

**Published:** 2024-05-02

**Authors:** Esther F. Afolalu, Thomas Salzberger, Linda Abetz-Webb, Stefan Cano, Rolf Weitkunat, Jed E. Rose, Christelle Chrea

**Affiliations:** 1 PMI R&D, Philip Morris Products S.A., Quai Jeanrenaud 5, 2000 Neuchâtel, Switzerland; 2 Institute for Statistics and Mathematics, WU Wien (Vienna University of Economics and Business), Welthandelsplatz 1, 1020 Vienna, Austria; 3 Patient-Centered Outcomes Assessments Ltd., 1 Springbank, Bollington, Macclesfield, Cheshire SK10 5LQ United Kingdom; 4 Modus Outcomes, St. James House, St. James Square, Cheltenham, GL50 3PR United Kingdom; 5 Department of Psychology, University of Fribourg, Rue P.-A.-de-Faucigny 2, 1700 Fribourg, Switzerland; 6 Rose Research Center, 7240 ACC Blvd., Raleigh, NC 27617 USA

**Keywords:** Psychology, Human behaviour, Outcomes research

## Abstract

How nicotine is administered has evolved from cigarettes to various delivery systems. Assessing perceived dependence on nicotine-containing products now requires accounting for product specificity while allowing comparisons across products and users. This study aims to develop a new self-report measure to assess perceived dependence on tobacco and nicotine products (TNPs) among exclusive and poly-TNP users. A draft version of the new measure, the ABOUT-Dependence, was constructed based on literature review, qualitative research, and expert opinion. Data for scale formation and psychometric assessment was obtained through a US-based web survey (n = 2334) that included additional dependence measures for convergent validity assessment. Qualitative research confirmed a preliminary conceptual framework with seven sub-concepts. Following a cognitive debriefing, 19 items were considered to best represent the different sub-concepts. Psychometric findings supported a three-domain structure [i.e., behavioral impact (five items), signs and symptoms (five items), and extent/timing of use (two items)] and an overall total composite score. The data confirmed convergent and known-group validity, as well as test–retest reliability. The ABOUT-Dependence is a 12-item, psychometrically sound, self-report measure that may be used as a tool for research and further understanding of perceived dependence across the spectrum of TNP and TNP users.

## Introduction

Studies on dependence on nicotine-containing products have primarily focused on cigarettes. With increased availability of multiple smoke-free tobacco and nicotine products (TNPs) (e.g. smokeless tobacco, e-cigarettes) that deliver nicotine in a less harmful manner, there is a clear need to develop alternative tools for assessing perceived dependence across a wide spectrum of products to better understand the public health impact of these smoke-free products (SFPs)^1–4^.

Leveraging self-reported symptoms as listed under the World Health Organization’s International Classifications of Diseases and Injuries (ICD) 10th Revision^5^ or the American Psychiatric Association’s Diagnostic and Statistical Manual Fifth Edition (DSM-5)^6^ to diagnose nicotine dependence, researchers have traditionally relied on self-report measures to assess perceived dependence on TNPs. Numerous measures have thus been developed, with the vast majority assessing perceived dependence in cigarette smokers^7–11^, including the very widely used Fagerström Test for Nicotine Dependence (FTND; now known as the Fagerström Test for Cigarette Dependence, FTCD)^12–14^. Such measures are not substitutes for clinical diagnoses of tobacco use disorder or nicotine dependence as defined in the DSM-5 and ICD-10 respectively, but they serve as key tools to support research on the use of TNPs.

Some of these measures have been adapted and validated for other TNPs, for example, the FTCD adapted for smokeless tobacco^15^. In contrast, other attempts have been made to develop non-cigarette-specific dependence measures for smokeless tobacco, e-cigarettes, and waterpipes^16–19^. Product-specific measures are required to consider product characteristics, including pharmacology, associated behaviors, and stimuli^1^. However, these measures do not allow product comparability, as the dependence scores derived from each product-specific measure are not necessarily based on the same metric. This raises the need for a measure to ensure appropriate comparability of the degree of perceived dependence across different TNPs. As poly-use (concurrent use of several TNPs) has become more frequent^20,21^, studies are now comparing levels of perceived dependence as a function of the number and type of product use^2,22,23^. This underscores the need to have a reliable and valid measure of perceived dependence for every product but also to assess the degree of self-reported dependence irrespective of the type and number of TNPs used.

There are two avenues toward achieving the goal of comparable measurement of perceived dependence among exclusive and poly-users: first, resort to existing measures and empirically derive their common core using quantitative methods; second, initiate a new scale development process starting from scratch and supported by qualitative and quantitative evidence. Both approaches have pros and cons. The first leverages what already exists, which may facilitate faster results. However, a possible limitation is that some items may not represent the concept of interest as they are selected from existing measures and do not stem from a dedicated conceptual framework. Even more problematic is the absence of relevant context-specific items to inform the conceptualization. In psychometrics, this issue is referred to as content validity, which is the cornerstone for any outcome measure development^24,25^. While the second approach is more time consuming because of its comprehensiveness, it ensures a better qualitative underpinning of the concept of interest and a closer match between the measure and conceptual framework. The first approach was recently used to compare the prevalence of well-known indicators of perceived dependence across different types of tobacco users^22^. Based on established cigarette dependence measures, Strong et al.^22^ derived a 16-item measure to enable perceived dependence comparison among cigarette, cigar, smokeless tobacco, e-cigarette, and hookah users. However, as noted by Morean et al.^26^, the proposed measure may have some limitations due to the lack of evidence that all items represent the concept of interest across all TNPs^26^. It is therefore worthwhile to develop a new scale that accurately reflects the evolving TNP landscape.

We undertook the development of a new self-report measure, the ABOUT-Dependence. It is part of the ABOUT Toolbox (Assessment of Behavioral OUtcomes related to Tobacco and nicotine products) initiative^27^, which promotes the use of best practice guidelines to develop fit-for-purpose self-report measures to assess perceptions and behaviors associated with the use of a wide range of TNPs. The present paper provides a detailed description of the multi-phase approach followed to develop and validate the measure.

### Basic conceptual foundation

We developed an initial conceptual framework based on a comprehensive literature review of theoretical contributions, empirical studies, and existing self-report questionnaires^13,14,28–34^. A literature search was conducted on EMBASE in February 2017 to identify existing self-reported measures and conceptualizations of dependence. In addition to the electronic search, 70 articles were identified manually. Articles and measures were selected for further review if they were relevant to tobacco or nicotine dependence. In total, twenty-five self-report measures^7–12,15–19,35–52^ and three interview-based assessments^22,53–55^ of perceived dependence were identified and reviewed. Supplementary Table S1 summarizes, for each of the 28 measures, the number of domains and items, the TNP for which it was initially developed and validated, and some key features of development and validation (namely, item generation, item reduction and psychometric analyses).

**Figure 1:**
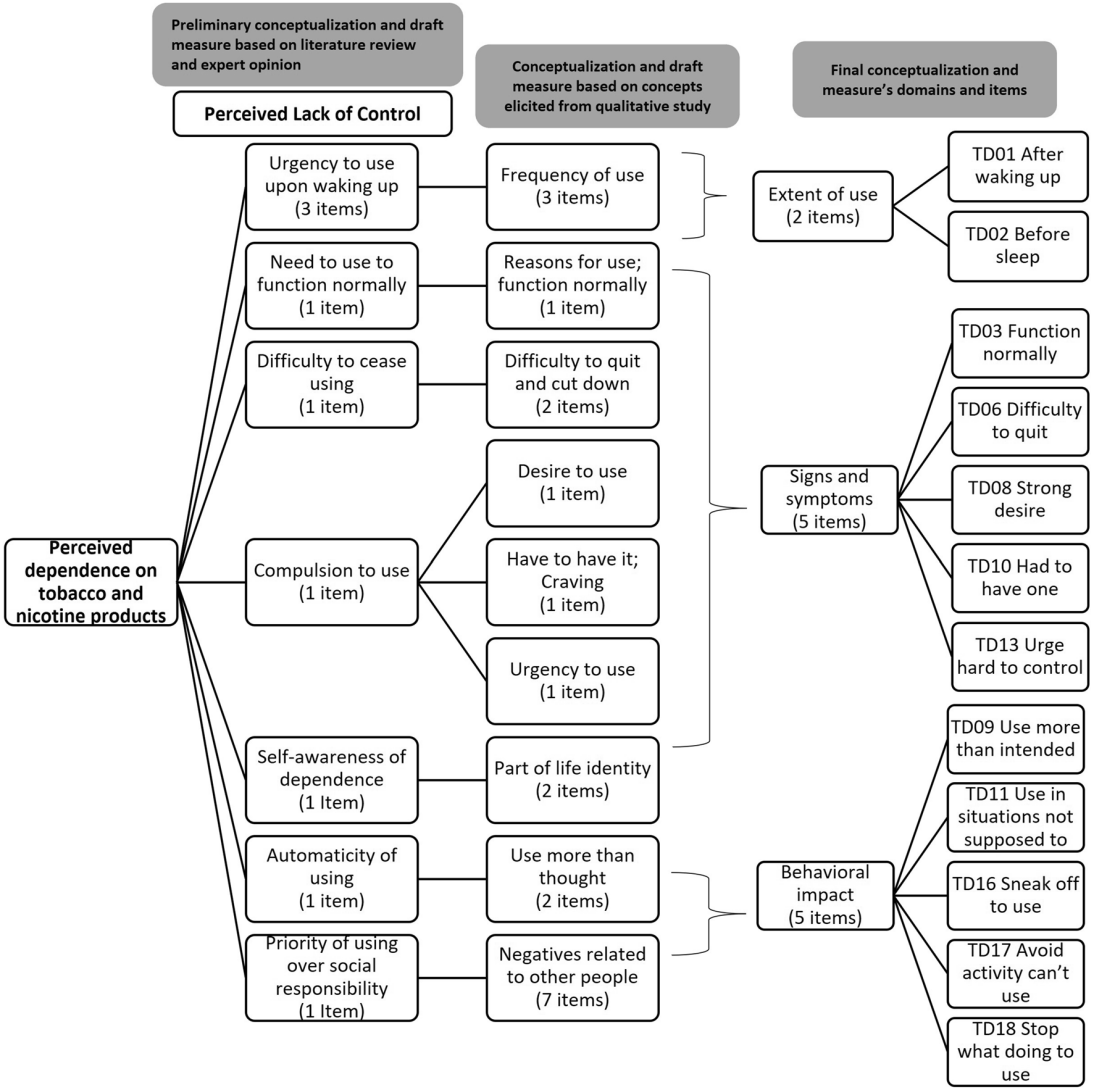
Evolution of conceptualization of perceived dependence for the ABOUT-Dependence Measure.

From these sources, *perceived lack of control* emerged as the core characteristic of perceived dependence, along with seven other sub-concepts as shown in the preliminary conceptualization (Fig. 1). Two experts in tobacco and nicotine addiction (JR and KF) and two experts in self-report measure development (TS and SC) reviewed the proposed conceptualization and contributed to the development of the first draft measure, which included nine items that characterized the degree of perceived dependence on three different response scales (intensity, frequency, or duration) adapted to the characteristics of the individual symptoms (see Supplementary Table S2). This preparatory work constituted the foundation for the subsequent qualitative and quantitative research to develop further and examine the psychometric properties of the new measure.

## Results

### Qualitative research

Table 1 presents the participants’ demographics. Over half (52%) of the sample were poly-TNP users.

**Table 1:** Overview of the sample demographics for both qualitative study and quantitative cross-sectional survey.

Characteristics	Qualitative study			Cross-sectional survey		
	Exclusive users n = 19	Poly-users n = 21	Total sample n = 40	Exclusive users n = 1181	Poly- users n = 1253	Total sample n = 2434
Age						
Mean (SD)	38.0 (14.95)	46.0 (11.06)	40.0 (27.0)	52.1 (13.9)	45.9 (13.0)	48.9 (13.8)
18–34 years, n (%)	7 (36.8)	7 (33.3)	14 (35.0)	155 (13.1)	305 (24.3)	460 (18.9)
35–49 years, n (%)	8 (42.1)	10 (47.6)	18 (45.0)	352 (29.8)	462 (36.9)	814 (33.4)
≥ 50 years, n (%)	4 (21.1)	4 (19.1)	8 (20.0)	674 (57.1)	486 (38.8)	1160 (47.7)
Gender, n (%)						
Female	7 (36.8)	8 (38.1)	15 (37.5)	442 (37.4)	532 (42.5)	974 (40.0)
Male	12 (63.2)	13 (61.9)	25 (62.5)	739 (62.6)	721 (57.5)	1460 (60.0)
Race, n (%)						
Black	4 (21.1)	8 (38.1)	12 (30.0)	66 (5.6)	148 (11.8)	214 (8.8)
White	12 (63.1)	8 (38.1)	20 (50.0)	1011 (85.6)	961 (76.7)	1972 (81.0)
Other^†^	3 (15.8)	5 (23.8)	8 (20.0)	104 (8.8)	144 (11.5)	248 (10.2)
Education level						
High school and below	6 (31.6)	8 (38.1)	14 (35.0)	189 (16.0)	141 (11.3)	330 (13.6)
Some college or college degree	5 (26.3)	7 (33.3)	12 (30.0)	459 (38.9)	507 (40.5)	966 (39.7)
Bachelor’s degree and beyond	8 (42.1)	6 (23.8)	14 (25.0)	533 (45.1)	605 (48.3)	1138 (46.8)
TNP currently used, n (%)						
Cigarette	5 (12.5)	17 (81.0)	22 (55.5)	250 (21.2)	932 (74.4)	1182 (48.6)
Cigars/cigarillos	4 (10.0)	9 (42.9)	13 (32.5)	250 (21.2)	529 (42.2)	779 (32.0)
E-cigarettes	5 (12.5)	13 (61.9)	18 (45.0)	252 (21.3)	775 (61.9)	1027 (42.2)
Smokeless tobacco	5 (12.5)	10 (47.6)	15 (37.5)	250 (21.2)	265 (21.1)	515 (21.2)
Pipe	0 (0.0)	0 (0.0)	0 (0.0)	47 (4.0)	161 (12.8)	209 (8.5)
Waterpipe	0 (0.0)	3 (14.2)	3 (7.5)	42 (3.6)	176 (14.0)	218 (9.0)
NRT	0 (0.0)	0 (0.0)	0 (0.0)	90 (7.6)	274 (21.9)	364 (15.0)
TNP use history						
Age start TNP use, mean^#^ (SD)	N/A	N/A	N/A	20.7 (9.1)	19.2 (6.0)	19.9 (7.7)
Current TNP/day, mean (SD)						
Cigarettes	14.6 (7.1)	8.6 (12.7)	10.0 (9.9)	12.5 (8.8)	9.9 (9.2)	10.5 (9.2)
Cigars/cigarillos	1.0 (1.3)	2.3 (3.0)	1.9 (1.5)	2.6 (5.5)	1.6 (3.1)	1.9 (4.1)
E-cigarettes (10 puffs)	1.77 (3.3)	3.9 (0.30)	3.5 (4.6)	11.7 (22.2)	4.2 (7.5)	6.0 (13.2)
Smokeless tobacco	3.6 (1.9)	1.1 (0.4)	2.0 (1.9)	4.4 (3.6)	2.6 (3.6)	3.5 (3.7)
Pipe	0.0 (0.0)	0.0 (0.0)	0.0 (0.0)	3.5 (4.1)	1.4 (3.0)	1.9 (3.4)
Waterpipe	0.0 (0.0)	0.3 (0.1)	0.1 (0.0)	0.8 (0.9)	1.3 (2.4)	1.2 (2.2)
NRT	0.0 (0.0)	0.0 (0.0)	0.0 (0.0)	6.3 (6.3)	2.3 (3.1)	3.3 (4.4)
Previous quit attempts, n (%)						
< 1 year	N/A	N/A	N/A	54 (4.6)	75 (6.0)	129 (5.3)
1–9 years	N/A	N/A	N/A	408 (34.5)	557 (44.5)	965 (39.6)
10 years and more	N/A	N/A	N/A	253 (21.4)	254 (20.3)	507 (20.8)
Missing	N/A	N/A	N/A	2 (0.2)	1 (0.1)	3 (0.1)
FTCD score*, n (%)						
Mild (score 0–3)	N/A	N/A	N/A	113 (45.2)	345 (37.0)	458 (38.7)
Moderate (score 4–6)	N/A	N/A	N/A	103 (41.2)	387 (41.5)	490 (41.5)
Severe (score 7–10)	N/A	N/A	N/A	34 (13.6)	200 (21.5)	234 (19.8)

^†^This included those who reported American Indian/Alaska native, Asian, Native Hawaiian or other Pacific Islander, Multiracial, or Some other race ^#^Study fieldwork was conducted in 2018 prior to change in legal age of using TNPs in US from 18 to 21 years old. *Fagerström Test for Cigarette Dependence adapted for different TNPs.

Concept elicitation data suggested several concepts directly linked to perceived dependence on a wide range of TNPs that largely confirmed the draft conceptualization developed in the early foundational work (Table 2). Key topics included amount of use, cravings, failure to quit, reasons for use, risky behaviors, or use despite negative consequences. Overall, there were no differences in concepts between poly- and exclusive users. However, when discussing amount and frequency of use, those who used e-cigarettes, pipes, or waterpipes reported they had greater difficulty quantifying the amount or times they used their products in a day than those who smoked cigarettes or used smokeless tobacco. Those who used cigarettes felt that the stigma of use was much greater than that of other TNPs, which corresponded with a greater likelihood of hiding or “sneaking” their use.

**Table 2:** Key themes and concepts elicited in the qualitative study.

Themes	Associated concepts
*Amount of use:* Perception that the amount they used their TNP(s) reflected, at least to some degree, their perceived level on dependence on their preferred product(s)	1. Number of years of use 2. Frequency of use 3. Quantity of use 4. Use more than the participant thought 5. Longest period without use 6. Increased use over time 7. Strength of product
*Cravings:* Described as a sense of anxiousness/hunger, once you have something in your head, then the craving keeps going until it is satisfied. It was considered an unpleasant feeling	1. Have to have/need to have 2. Urgency to use 3. Cravings/curbs cravings 4. Desire to use
*Failure to quit*: Trying to quit smoking was considered to be very hard by respondents; once someone had started, it was very hard to stop. Respondents who considered quitting to be difficult were more likely to be those who had already tried and failed rather than those who considered it to be easy but had yet to try quitting	1. Trying, succeeding, failing 2. Difficult to quit 3. Desire to quit 4. Psychological factors (part of life/identity, do not want to quit) 5. Other factors (withdrawal symptoms, cessation aids felt as non-efficient)
*Reasons for use/non-use:* One of the key reasons was to use it as a coping mechanism, either because of stress, boredom, etc	1. Reduce stress 2. Time for self 3. Increase alertness
*Risky behaviors or use despite negative reasons to use TNPs:* An acknowledgment that using a TNP was not good for them, but they continued their use despite the health risks and the social consequences	1. Negatives related to other people 2. Product issues 3. Risky beliefs 4. Regret

TNP, Tobacco and nicotine product.

Cognitive debriefing interviews with the first 20 participants in Wave 1 led to the removal of a problematic item focused on the “proportion of available time” the person used the product because participants had difficulty conceptualizing and estimating the idea of available time to use their product. Revisions were also made to some items and response options through the two waves of interviews to enhance comprehension. In total, 11 items that were specifically elicited by the participants in the concept elicitation part were added to the draft measure (see Supplementary Table S2 for more details on concepts, their definitions, related items, and examples of indicative quotes from participants). Incorporation of the findings from the qualitative research and review by the four experts (SC, TS, KF, and JR) resulted in an updated conceptualization (see Fig. 1) and 19-item revised draft measure for the psychometric assessment carried out in subsequent quantitative research.

### Quantitative research

Table 1 presents the participants’ demographics, highlighting the appropriate implementation of quotas in the study.

Outcomes of the Rasch Measurement Theory (RMT) analyses against the acceptability criteria outlined in Supplementary Table S3 are summarized in Table 3. All but one participant completed all items in the measure, which supports acceptability. The initial psychometric analyses revealed some problems in the 19-item draft measure; in particular, thresholds for 3 items were disordered, 13 out of 19 items were found to have fit residuals out of the recommended guidelines (this was solely due to the large sample size as graphical inspection did not reveal any hint of unequal item discrimination), and 9 pairs of items had residual correlations > 0.30, which indicated local dependence and thus redundancy. Importantly, RMT analyses suggested that the 19-item measure did not form a strictly unidimensional set. Further item deletion or combining items into subtests to account for response dependency did not result in statistical indicators demonstrating a strictly unidimensional scale. As a consequence, perceived dependence was reconceptualized (see Fig. 1), based on a discussion with the four experts, as a multidimensional construct including three domains: *Extent of Use*, covering the timing, urgency, or pervasiveness with which the product(s) is/are used; *Behavioral Impact*, comprising aspects of how perceived dependence impacts daily activities; and *Signs and Symptoms* of perceived dependence experienced by TNP users.

**Table 3:** Psychometric Performance Summary (Rasch Measurement Methods [RMT]).

Proposed scale	Disordered thresholds	Distribution of item thresholds	Floor/ceiling effects	Peak of information plot	PSI	Item fit residual	Item χ^2^	Person fit residual	Local dependence	Differential item functioning (DIF)
	# of items/ total # of items (%)	Description (% Coverage)^a^	Description; n/N (%)	Description; approx. location)^b^	Value	# of items outside ± 2.5/total # of items (%)^c^	# of items significant/total # of items (%)^d^	# of persons outside ± 2.5/total # of persons (%)	# of pairs of item residual correlations > 0.30 of the avg./total # of pairs^e^	# of items significant/total # of items (%)^f^
Initial 19-items	3/19 (16)	Good (92)	F + C; 37/2434 (2)	Center; 0	0.95	13/19 (68)	6/19 (32)	293/2434 (12)	9/171	1/19 (5)
Behavioral Impact	0/5 (0)	Reasonable (86)	F + C; 310/2434 (13)	Less, − 0.2	0.81	4/5 (80)	0/5 (0)	106/2434 (4)	0/10	0/5 (0)
Signs and Symptoms	0/5 (0)	Reasonable (90)	F + C; 192/2434 (8)	Less; − 0.4	0.89	3/5 (60)	0/5 (0)	82/2434 (3)	0/10	0/5 (0)
Extent of Use	0/2 (0)	Reasonable (80)	F + C; 350/2434 (14)	Less, − 0.2	0.73	0/2 (0)	0/2 (0)	176/2434 (7)	n.a	0/2 (0)
ABOUT-Dependence Index	1/6 (17)	Reasonable (90)	F + C; 47/2434 (2)	More; + 0.2	0.82	6/6 (100)	0/6 (0)	103/2434 (4)	0/15	0/6 (0)

χ^2^, Chi-square; C, Ceiling effect; F, Floor effect; n.a., not applicable; PSI, Person separation index.

^a^Distribution of Item Thresholds is presented in % of coverage and completed by a qualitative description based on arbitrary cut-offs; *good* indicates coverage of the item thresholds was > 90%, *reasonable* indicates coverage of the item thresholds was 70%-90%, *poor* indicates coverage of the item thresholds was < 70%.

^b^Peak of Information Plot is presented in logits unit based on the Person Threshold distribution and completed by a qualitative description: *More* suggests that the scale targets participants with a higher perceived dependence.

^c^This was to be expected given the large sample size but should still be a helpful indicator if/when some items are much worse fitting relative to others.

^d^In the statistical assessment, the actual n was adjusted to 500 to mitigate excessive power and parallel fit assessment based on a sample size of 500, deemed appropriate for the present psychometric analysis.

^e^Residual correlation for extent of use not informative due to only two items.

^f^ Listed is DIF by TNP with adjusted n = 500.

Six items were removed from the 19-item draft measure based on conceptual redundancy (local dependence), while one was omitted based on psychometric misfit (see Supplementary Table S4 for item reduction details. The reduced 12-item measure retained full content coverage of perceived dependence, and the three domains showed good psychometric performance according to RMT criteria (Tables 3 and 4).

**Table 4:** Final item calibration and fit (Rasch measurement methods [RMT]).

Domain/item	Perceived dependence	Item location	SE	Item fit χ^2^ (df, p)^a^
Behavioral impact				
TD09 use more than intended	Less dependence | | | | | More dependence	− 0.86	0.03	14.4 (9, 0.11)
TD18 stop what doing to use		− 0.23	0.03	15.4 (9, 0.08)
TD11 use in situations not supposed to		0.14	0.03	6.8 (9, 0.66)
TD16 sneak off to use		0.31	0.03	10.6 (9, 0.30)
TD17 avoid activity can’t use		0.63	0.03	22.2 (9, 0.01)
Signs and symptoms				
TD08 strong desire	Less dependence | | | | | More dependence	− 0.78	0.04	11.4 (9, 0.25)
TD06 difficult to quit		− 0.52	0.03	3.1 (9, 0.96)
TD10 had to have one		0.12	0.03	10.5 (9, 0.31)
TD03 function normally		0.33	0.03	5.8 (9, 0.76)
TD13 urge hard to control		0.85	0.03	7.3 (9, 0.60)
Extent of use				
TD01 after waking up	Less dependence | More dependence	0.04	0.02	12.5 (8, 0.13)
TD02 before sleep		− 0.04	0.03	4.6 (8, 0.80)
ABOUT-dependence index				
Extent of use—e*xclusive users of e-cigarette*	Less dependence | | | | | | | | | More dependence	− 0.31	0.03	1.9 (9, 0.99)
Signs and Symptoms		− 0.11	0.01	20.2 (9, 0.02)
Extent of use—*exclusive users of cigarettes, smokeless tobacco, pipes or NRT*		− 0.05	0.02	3.1 (9, 0.96)
Extent of use—*poly-users*		0.02	0.01	4.2 (9, 0.90)
Extent of use—*exclusive users of waterpipe or cigars*		0.10	0.04	2.4 (9, 0.98)
Behavioral impact		0.35	0.01	6.4 (9, 0.69)

TD, refers to the measure’s item number; SE, standard error of location; χ^2^, chi-square; df, degrees of freedom; p, p value; NRT, nicotine replacement therapy;

^a^In the statistical assessment the actual n was adjusted to 500, which is deemed appropriate for the present Rasch psychometric analysis to mitigate excessive power.

*Suitability of the response format and targeting:* Response option thresholds were properly ordered, suggesting a meaningful response format. In terms of targeting, person measurements were well covered (80–90%).

*Item hierarchy:* Regarding the *Behavioral Impact* domain, we expected some order relations between the items based on conceptual considerations and qualitative research findings. Being a basic manifestation of perceived dependence, “using more of the product than intended” should be most easily endorsed. Endorsing “stopping an activity to use a TNP” was supposed to require less perceived dependence compared to avoiding an activity altogether. For the *Signs and Symptoms* domain, “a strong desire” was supposed to be more readily endorsed than finding it “hard to control the urge,” as the latter implies the former but not vice versa. The RMT analysis confirmed these expected relationships. See Table 4 for item calibration, location, and fit statistics for all 12 items within each domain.

*Reliability:* Person separation indices (PSIs) for all three domains were satisfactory (0.73 to 0.89) with the *Signs and Symptoms* domain having a slightly higher reliability compared to the other domains.

*Item invariance:* For all three domains, item invariance, crucial for comparability of measurements, was supported in that there was no differential item functioning (DIF) by type of user (exclusive vs. poly) or key sociodemographics (age, sex, education).

**Figure 2:**
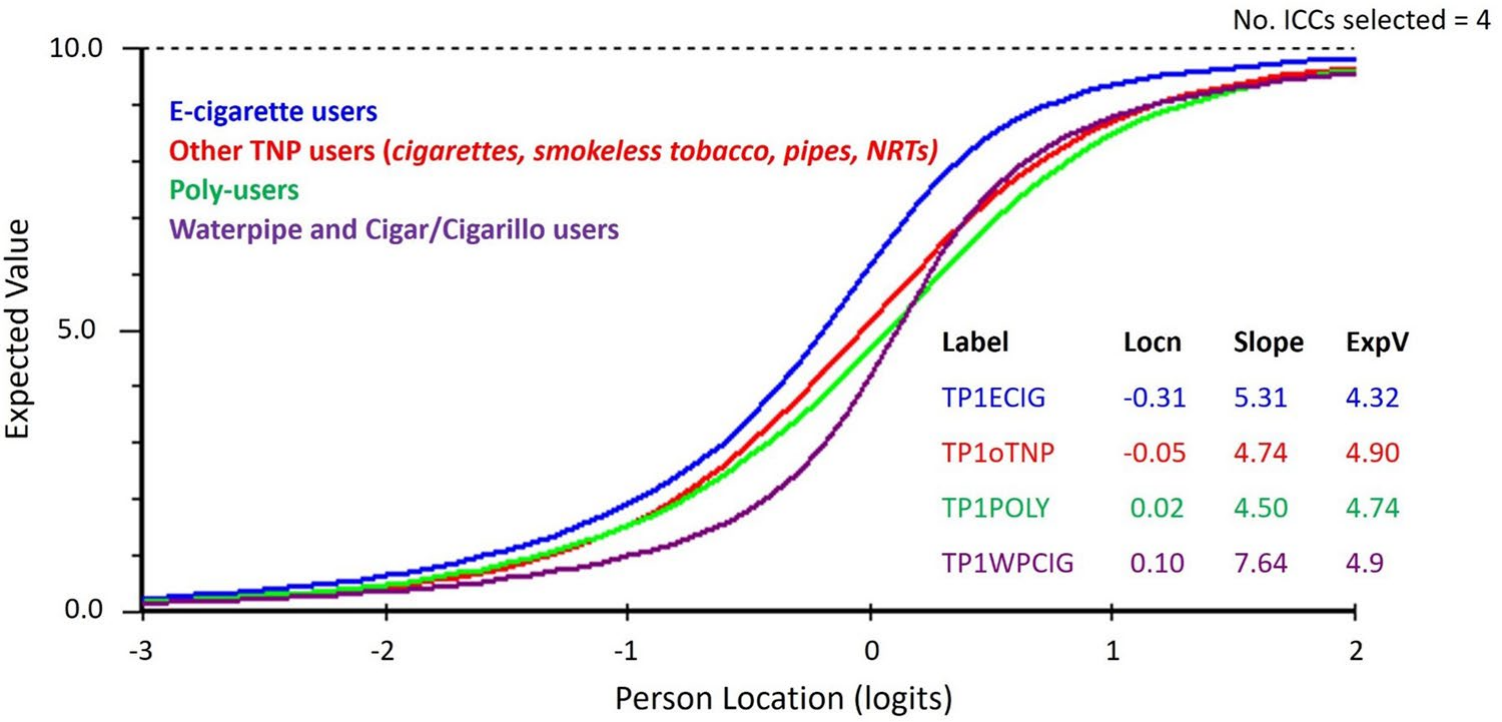
Item characteristic curves (ICC) for the extent of use domain across different tobacco and nicotine products (TNPs) once the item (subtest) was split to account for Differential Item Functioning (DIF). DIF was accounted for by estimating separate subtest parameters for some TNPs.

*Unidimensionality:* Separately, all three domains were unidimensional. As the three domain measures were correlated in the order of 0.5 to 0.8 in the entire sample, the ABOUT-Dependence index (composite score) capturing overall perceived dependence was formed covering all three domains, to be considered in addition to the individual scale scores. However, the relationship of the *Extent-of-use* domain and the two remaining domains varied by TNP and user type. Given the same dependence, DIF was noted, as e-cigarette users reported a greater extent of use, waterpipe and cigar users reported a lower extent of use, and other TNPs (cigarettes, smokeless tobacco, pipes, nicotine replacement therapy [NRT]) were very similar regarding the extent of use (Fig. 2). The DIF was accounted for by estimating separate subtest parameters for some TNPs.

*Classical Test Theory (CTT) criteria* Table 5 provides a summary of the CTT analyses (according to the criteria defined in Supplementary S5), supporting the psychometric validity and reliability of the item-reduced measure. Moreover, Pearson and intra-class correlations showed very good test–retest reliability (> 0.70).

**Table 5:** Scaling assumptions (classical test theory [CTT]).

Item	N	Scaling assumptions				
		Possible range (mid-point)	Actual score range	Mean score	Variance	CITC
Behavioral impact						
TD09 use more than intended	2434	1–5 (3)	1–5	2.8	1.3	0.64
TD11 use in situations not supposed to	2433	1–5 (3)	1–5	2.2	1.5	0.72
TD16 sneak off to use	2434	1–5 (3)	1–5	2.1	1.5	0.76
TD17 avoid activity can’t use	2434	1–5 (3)	1–5	1.9	1.4	0.79
TD18 stop what doing to use	2434	1–5 (3)	1–5	2.4	1.6	0.77
Signs and symptoms						
TD03 function normally	2434	1–5 (3)	1–5	3.0	1.7	0.76
TD06 difficult to quit	2434	1–5 (3)	1–5	3.4	1.9	0.75
TD08 strong desire	2434	1–5 (3)	1–5	3.4	1.1	0.80
TD10 had to have one	2434	1–5 (3)	1–5	3.0	1.4	0.82
TD13 urge hard to control	2434	1–5 (3)	1–5	2.7	1.6	0.78
Extent of use						
TD01 after waking up	2434	1–6 (3.5)	1–6	3.5	2.7	0.71
TD02 before sleep	2434	1–6 (3.5)	1–6	3.5	2.3	0.71

TD, refers to the measure’s item number; N, number of cases; CITC, Corrected Item-total correlation test.

*Convergent validity* The validity of the new measure was supported by moderately high correlations with existing product-specific perceived dependence measures among exclusive users (Table 6).

**Table 6:** Convergent validity and correlations between ABOUT–Dependence and existing dependence measures.

TNP	Measure	n	Spearman correlations			
			Behavioral impact	Signs and symptoms	Extent of use	ABOUT-dependence index
Cigarette	FTCD for cigarettes	250	0.46	0.64	0.79**	0.73*
	WISDM-brief version	248	0.61	0.75*	0.60	0.81**
	CDS-5	243	0.38	0.71*	0.72**	0.70
E-Cigarettes	PS-ECDI	251	0.45	0.65	0.71**	0.71**
	CDS-5 adapted for e-cigarettes	242	0.37	0.63	0.68**	0.65*
Smokeless tobacco	FTCD for smokeless tobacco	250	0.40	0.55	0.74**	0.65*
	CDS-5 adapted for smokeless tobacco	238	0.46	0.78**	0.68	0.78**
Cigar/cigarillos	FTCD adapted for cigars/cigarillos	248	0.58*	0.56	0.69**	0.66*
	CDS-5 adapted for cigars/cigarillos	224	0.71	0.73**	0.71	0.80**
Waterpipe	LWDS-11	42	0.59*	0.58	0.48	0.65**
	CDS-5 adapted for waterpipe	42	0.45	0.58**	0.32	0.54*
Pipe	CDS-5 adapted for pipe	45	0.65	0.72*	0.61	0.76**
NRT	CDS-5 adapted for NRT	86	0.21	0.59**	0.43*	0.43*

CDS, Cigarette Dependence Scale; FTCD, Fagerström Test for Cigarette Dependence; LWDS, Lebanon Waterpipe Dependence Scale; NRT, Nicotine Replacement Therapy; PS-ECDI, Penn State Electronic Cigarette Dependence Index; NRT, Nicotine Replacement Therapy; TNP, Tobacco and Nicotine Product; WISDM, Wisconsin Index of Smoking Dependence Motives.

Correlations: < 0.30 reflect poor evidence, correlations between 0.3 and 0.5 reflect moderate evidence, correlations between 0.5 and 0.7 (*) reflect sufficient evidence, and correlations > 0.7 (**) reflect very good convergent validity.

*Known group validity:* As expected, domain scores differed by key product use patterns. Very light users (< 5 units per day) scored lowest on the ABOUT-Dependence index, while heavy (20 + units per day) users scored highest. While there was no difference between light (5–10 units per day) and moderate (10–20 units per day) users (Scheffé *post-hoc* test, p = 0.17), all other pairwise comparisons showed significant differences in the expected direction (Scheffé *post-hoc* tests, all p < 0.001). These results are consistent with previous findings that greater TNP consumption is associated with higher perceived dependence^56^. Poly-users reported higher perceived dependence than exclusive users (t = 15.7, df = 2430, p < 0.001), which is also in line with previous studies^22,57^. A difference in the amount of total TNP use partly explains the difference. However, including total use as a covariate still showed a significant difference between exclusive and poly-users (one-way analysis of variance F = 169.1, df1 = 1, df2 = 2431, p < 0.001 for user type).

*Perceived dependence across product types:* Scores by type of TNP for exclusive users in the study (Table 7) showed that cigarette smokers reported higher perceived dependence scores, followed by users of NRT, e-cigarettes, smokeless tobacco, waterpipe, pipes, and cigars/cigarillos.

**Table 7:** ABOUT-Dependence measure score of perceived dependence across different products and user types.

User type	TNP	n	Measure score in the 0-to-100 metric Mean (SD)			
			Extent of use	Signs and symptoms	Behavioral impact	ABOUT-dependence index
Exclusive users	Cigarettes	250	54.9 (18.7)	56.8 (18.7)	37.7 (18.5)	54.0 (10.1)
	E-Cigarettes	252	58.5 (26.8)	50.5 (19.5)	36.7 (20.0)	51.3 (11.8)
	Smokeless tobacco	250	47.0 (23.6)	51.8 (20.7)	34.2 (19.1)	50.6 (11.3)
	Cigars/Cigarillos	250	23.1 (25.0)	28.5 (23.1)	18.9 (20.2)	34.9 (17.8)
	Waterpipe	42	32.1 (25.0)	40.3 (20.5)	34.4 (21.7)	45.9 (14.7)
	Pipes	47	31.3 (22.0)	33.0 (20.5)	21.3 (17.6)	38.3 (15.8)
	NRT	90	53.5 (22.4)	55.3 (15.0)	35.1 (19.5)	53.2 (8.7)
Poly-users	≥ 2 TNPs	1253	54.7 (23.0)	59.1 (20.6)	45.0 (21.3)	56.3 (12.1)

NRT, Nicotine replacement therapy; TNP, Tobacco and nicotine product.

*Scoring and estimation of measures*: To facilitate appropriate use of the ABOUT-Dependence measure, a calibrated scoring scheme was developed to enable conversion of unweighted sum scores into linear measures based on weighted likelihood estimation (WLE)^58^, and the calibration was done with the unrestricted Rasch model for polytomous responses^59,60^. For complete data, the resulting conversion table transfers sum scores to logit measures, which are mapped to a 0–100 scale for convenience. Depending on the research question and focus of interest, the measure can be scored and interpreted using separate scores for each domain and/or total ABOUT-Dependence index across the three domains in the measure^61^. Full details and information on the access, use, and scoring of the measure are provided through eProvide by Mapi Research Trust (https://eprovide.mapi-trust.org/).

While the new measure is primarily meant for comparative measurement across TNPs, it can also be used for exclusive product use. As we expect existing product-specific measures to remain in use, to ensure comparability of research findings over time, it is important to link the new measure and its metric to existing scales. Salzberger et al. provide further information on how various existing measures (e.g., FTCD) can be linked to the newly established metric from the ABOUT-Dependence^62^. This equating procedure establishes crosswalks that provide the opportunity to transform raw scores on various existing product-specific measures to one another and to a common metric established by the new measure.

## Discussion

Psychometric performance of the new ABOUT-Dependence measure was strong across both RMT and CTT analyses, supporting the conclusion that the 12-item measure, consisting of a 2-item *Extent of Use* scale, a 5-item *Behavioral Impact,* and a 5-item *Signs and Symptoms* scale, is a psychometrically reliable and valid self-report measure. In addition, a composite ABOUT-Dependence Index can be formed as a summary measure across all three scales. Compared to the individual scales, the index exhibited even smaller floor and ceiling effects.

There are four key strengths of this new measure. First, the content validity was evidenced by comprehensive information gathered from a literature review, expert opinions, and concept elicitation and cognitive debriefing interviews with TNP users in line with best practices and guidelines^63^. Such a comprehensive approach has not always been applied consistently in the development and validation strategies of TNP self-report dependence measures. For example, as identified in the literature review, the development of existing measures varied widely, with TNP users’ involvement in the item generation and reduction only sporadically included.

Second, the diversity of subpopulations of TNP users sampled in the studies provided a broad frame of reference that demonstrated the validity of the measure. In total, seven TNP categories were explored, allowing for the assessment of measurement comparability across a large spectrum of TNPs and providing a solid foundation for the measure to be used with other products (e.g., heated tobacco products and nicotine pouches).

Third, the fit of the data to the Rasch measurement model and the lack of DIF by TNP for the *Behavioral Impact* and *Signs and Symptoms* domains demonstrate that these items worked as a set, representing manifestations of unidimensional experienced symptoms and perceived burden of dependence, respectively, for diverse products. At the same time, the presence of DIF by product type for the *Extent of Use* scale as part of the composite variable may be linked to the high variability in urgency and frequency of use when considering products such as cigarettes and e-cigarettes versus waterpipes and cigars.

Finally, compared to existing measures, the new measure provides a more interpretable measure of perceived dependence by referring to the hierarchy of individual items and their response categories reflected in their locations on the continuum of perceived dependence. Thus, for each level of perceived dependence, the most likely response to each item provides a detailed characterization of the degree of dependence perceived by a respondent. In addition, the ABOUT-Dependence index allows for consolidation of the ABOUT-Dependence measure and existing product-specific measures by providing crosswalks that bridge the metrics of different measures^62^.

There are also some limitations to our development. First, when examining scores across products with the ABOUT-Dependence measure, it is important to realize that it assesses the perception of existing perceived dependence on a specific TNP or—in the case of poly-users—a bundle of TNPs. In terms of the findings on perceived dependence estimates by TNP type in this study, caution should be exercised when interpreting the estimates as population statistics. This study’s purpose was scale development; it was not designed to provide general population estimates of perceived dependence on different TNPs, nor can it indicate the physiological addiction or abuse liability of TNPs. Second, while the implementation of quotas per TNP type and key sociodemographics aimed at broad coverage of the population and possible applications of the measure, an adequate representation was not fully achieved for products such as NRTs. When looking at the small NRT user subgroup, only one NRT user was recruited in the qualitative study, limiting the assessment of content validity of the measure in this group. In addition, in the quantitative study, it appears that this group may not be representative of exclusive NRT users and those who use NRTs as intended; some were long-term users of NRTs with no intention to quit, and some reported occasional use of other TNPs during the past 12 months. This could explain why the level of perceived dependence of NRT users was not very different from what was observed in cigarette smokers. This clearly calls for further research, particularly in different categories of NRT users, to better contextualize the interpretation of perceived dependence scores and further validation of the measure. In addition, it is worth noting that in the quantitative research, the GkF KnowledgePanel^®^ utilized probability-based sampling aimed to be representative of the US population and whilst this was fairly reflected in the sample of respondents in terms of age, gender, race, and educational attainment^64^, greater diversity of the sample could have further supported the universal applicability of the measure across different demographics and the broader population of TNP users.

Finally, it is worth noting that Strong et al.^22^ also demonstrated that a 16-item measure of perceived dependence adapted for use with specific TNPs identified a common dimension perceived of dependence that could be used to assess variability in perceived dependence across different TNP users. Nevertheless, the ABOUT-Dependence measure has a broader conceptual coverage, especially since it captures the domain of behavioral impact, which is notably missing from Strong et al.’s measure but was shown in the current findings to be an important aspect of perceived dependence. The behavioral impact domain may be worth investigating further to better understand the effect of perceived dependence on different TNPs on behavioral aspects of daily life.

In all, based on a conceptual framework of perceived dependence, the interplay of insights from qualitative research, and psychometric analysis, a new three-domain measure of perceived dependence could be empirically supported. Applied longitudinal studies will further reveal how perceived dependence may evolve over time and the predictive validity of the measure with regard to other TNP use behaviors^65^. Additional cross-validation studies are also necessary to provide further evidence of the utility of the new measure to advance the understanding and interpretation of self-reported measurement of dependence across the whole spectrum of TNPs, user types, and cultural contexts. The measure could be used to provide additional standardized assessment and data for use in clinical research and practice to better understand impact of the use of multiple and different TNPs, the psychological and behavioral aspects of tobacco and nicotine dependence and evaluate the effectiveness of related clinical harm reduction or cessation interventions. With the outlook of increasing use of the ABOUT-Dependence in tobacco and nicotine research studies, we envision a rapidly expanding knowledge base to support tobacco harm reduction and help inform regulators and health and public policy communities when assessing the potential public health impact of SFPs.

## Methods

### Qualitative research

#### Design overview

The qualitative research served the purpose of expanding our conceptual understanding of perceived dependence in TNP users and examining to what extent the instructions and item content of the initially drafted measure were appropriate, relevant, comprehensive, and understandable to different types of TNP users. The research comprised two waves of concept elicitation and cognitive debriefing interviews (August 2017) and consultations with the same four subject matter experts from the foundational work (September 2017).

#### Study population and recruitment process

The research was performed in accordance with relevant guidelines/regulations, including the Declaration of Helsinki. Forty individuals (20 exclusive users and 20 poly TNP users) in the United States (US) participated in an open-ended concept elicitation interview combined with cognitive debriefing. Participants were recruited through a proprietary database maintained by the market research company AOC Marketing Research (Charlotte, NC, US). The sample was purposively recruited to produce diversity in terms of characteristics such as sex, age, race, educational attainment, and a balanced representation of TNPs (cigarettes, cigar/cigarillos, e-cigarettes, and smokeless tobacco) among exclusive TNP users. Participants received USD $100 as compensation for their participation.

The main inclusion criteria for eligibility in the study were participants to be of legal age of smoking or above and a regular current TNP user who have used their product at least 100 times in their lifetime and used their product at least once in the past 7 days. The product use criteria were based on existing classifications^66,67^ for current TNP users and to ensure participants were using their product at the time of the study. The main exclusion criteria were self-reported use of illegal drugs in the last 12 months; participation in a market research study in the past 6 months; and employment in certain sectors related to advertising, media, public relations, or marketing; a company that manufactures tobacco and nicotine products; or healthcare, government, and clinical organizations.

#### Interview procedure

The study was carried out in two waves over the course of 4 days. In the first 2 days (Wave 1), 20 interviews were conducted. Following some modifications to the measure based on the first 20 interviews, a second wave of 20 interviews was executed similarly (Wave 2).

Each interview was divided into two parts and followed a semi-structured interview guide. In the first part, concept elicitation techniques were used to identify aspects of perceived dependence relevant to different TNP users. This was geared toward developing new items to supplement the initial nine-item draft measure. In the second part of the interview, cognitive debriefing was used to ensure relevance, clarity, and ease of completion of the previously generated nine items. In Wave 2, the draft supplementary items were developed based on the Wave 1 concept elicitation. A “think aloud” process^68,69^ was followed where participants were asked to complete the items while saying out loud what they were thinking, and specifically to note any problematic areas (instructions, items, rating scales) and suggest ways to improve the measure. Interviews were audio-taped and transcribed verbatim.

#### Data analysis: concept elicitation

Transcripts were analyzed thematically using Microsoft Excel (Seattle, WA, US) and MAXQDA software (VERBI, Berlin, Germany; https://www.maxqda.com/). Participant-generated concepts were inductively categorized into concepts of the initial conceptualization. Saturation (the point at which no new information is arising from the qualitative data) was also assessed by comparing concepts arising in the first five interviews to those emerging in the next five interviews, for a total of eight sets of data to compare. If no new information arose in the eighth set, saturation was said to be achieved.

#### Data analysis: cognitive debriefing

The analysis aimed to identify wording ambiguities and assess the relevance and acceptability of each item, the response scale, and the instructions. Additional items suggested by Wave 1 participants that could further expand the measurement of perceived dependence were also explored and tested with participants of Wave 2.

### Quantitative research

#### Design overview

The psychometric evaluation study was designed as a cross-sectional, two-wave, internet-based survey with purposive stratified sampling of legal age TNP users in the US. Participants who completed the first survey (Wave 1; Baseline) were invited to complete a second similar but shorter survey 7–10 days later to determine the test–retest reliability of the new measure (Wave 2; 7–10-day follow-up).

The sampling provided for quotas in terms of TNP use behavior (50% exclusive and 50% poly-users). Exclusive TNP users were equally split into cigarette, smokeless tobacco, e-cigarette, cigars/cigarillos, and other TNPs (pipe, waterpipe, and NRTs). At least 40% of TNP poly-users had to smoke cigarettes. In addition, quota sampling based on age, sex, and education was applied to the intended total sample size of 2500 for Wave 1^70^. The target sample size for the Wave 2 follow-up survey was 1000 completers (500 exclusive TNP users and 500 poly-TNP users). The survey fieldwork took place from April to May 2018.

#### Study population and recruitment process

The research was performed in accordance with relevant guidelines/regulations, including the Declaration of Helsinki. Participants were identified via the proprietary Growth from Knowledge (GfK, Nuremberg, Germany) consumer online panel KnowledgePanel®, which aimed to be representative of the US population. The same inclusion and exclusion criteria used in the qualitative research were also applied here. In terms of product use specifically, exclusive TNP users should self-report having used only one product at least 100 times in their lifetime and have used this product at least once in the past 7 days and poly-users should self-report as current users of at least two TNPs, have used at least each of the products 100 times in their lifetime, and have used each at least once in the past 7 days. Participants were compensated for their participation according to the GfK KnowledgePanel^®^ points systems incentive program in which respondents are credited points which can be redeemed for cash or other rewards for completing surveys.

#### Measures and survey procedure

Upon participants’ enrollment in the study, the cognitively debriefed draft version of the measure was administered first. Depending on the TNP(s) poly-user participants declared using in the last 7 days at the time of the survey, they were administered additional product-specific dependence measures for assessment of convergent validity: FTCD for cigarette and cigar users^12^, smokeless tobacco FTCD (SLT-FTCD) for users of smokeless tobacco products^16^, the Penn State e-cigarette dependence index (PS-ECDI) for e-cigarette users^18^, the Lebanon Waterpipe Dependence Scale (LWDS) for waterpipe users^19^; the cigarette dependence scale (CDS) for cigarette smokers or an adaptation for users of other TNPs^7^, and the brief Wisconsin Inventory of Smoking Dependence Motives (WISDM) for cigarette smokers^46^. A shorter but otherwise similar survey to Wave 1 was administrated at Wave 2 (7 to 10 days after Wave 1).

#### Data analysis

*Item reduction and internal construct validity:* Assessed through the application of the RMT analyses^71^ aiming at comparing observed data against the stringent criteria of the Rasch model (see Supplementary Table S3 for more details on the definition and acceptability criteria for RMT analysis). Analyses rooted in CTT^72^ were also conducted on the item-reduced scales to provide a comprehensive psychometric assessment (see Supplementary Table S5 for more details on the definition and acceptability criteria for CTT analysis).

*Test–retest reliability:* Pearson and intra-class correlations were calculated between the scores of the item-reduced measure at Wave 1 and Wave 2.

*Convergent validity:* Spearman correlations between the item-reduced measure and the existing product-specific dependence measures.

*Known group validity:* The following group comparisons were conducted: heavy (≥ 20 units/day) vs. moderate (10–20 unit/day) vs. light (5–10 units/day) vs. very light users (less than 5 units/day) (as the number of units consumed per day increases, perceived dependence was expected to increase)^56^; poly- versus exclusive users (with level of perceived dependence in poly-users expected to be higher)^22,26^.

RMT analyses were conducted using RUMM2030 (RUMM Laboratory Pty Ltd., Duncraig, Australia; https://www.rummlab.com.au/rumm-2030)^73^, and CTT analyses, test–retest, known group, and convergent validity analyses were conducted using IBM SPSS 21 (IBM Corp., Armonk, NY, US)^74^.

### Ethics approval and consent to participate

The qualitative study was approved by the New England Institutional Review Board (IRB) (NEIRB# 120170184) on July 12, 2017, and written informed consent was obtained from participants. The quantitative study was approved by the New England IRB (NEIRB# 120180022) on March 14, 2018, and electronic informed consent was obtained from participants.

### Supplementary Information

Supplementary Tables.

## Data Availability

The datasets generated and/or analyzed during the current study available from the corresponding author on reasonable request.

## References

[1] Fagerström K, Eissenberg T (2012). Dependence on tobacco and nicotine products: A case for product-specific assessment. Nicotine Tob. Res..

[2] Strong DR (2015). Measurement of multiple nicotine dependence domains among cigarette, non-cigarette and poly-tobacco users: Insights from item response theory. Drug Alcohol Depend..

[3] Fagerström K (2018). A comparison of dependence across different types of nicotine containing products and coffee. Int. J. Environ. Res. Public Health.

[4] Halpern-Felsher, B., Kim, H. & Weaver, S. What do we need to develop better measures of e-cigarette use, dependence, perceptions and policy? Transdisciplinary Topical Discussions #4. In *23rd Annual Meeting of the Society for Research on Nicotine & Tobacco *(2017).

[5] World Health Organization (WHO). *International Statistical Classification of Diseases and Related Health Problems 10th Revision* <https://www.who.int/classifications/icd/ICD10Volume2_en_2010.pdf> (2010).

[6] American Psychiatric Association. *Diagnostic and Statistical Manual of Mental Disorders* (2013).

[7] Etter JF, Le Houezec J, Perneger TV (2003). A self-administered questionnaire to measure dependence on cigarettes: The cigarette dependence scale. Neuropsychopharmacology.

[8] Shiffman S, Waters A, Hickcox M (2004). The nicotine dependence syndrome scale: A multidimensional measure of nicotine dependence. Nicotine Tob. Res..

[9] Wellman RJ (2005). Measuring adults' loss of autonomy over nicotine use: The hooked on nicotine checklist. Nicotine Tob. Res..

[10] Piper ME (2004). A multiple motives approach to tobacco dependence: The Wisconsin inventory of smoking dependence motives (WISDM-68). J. Consult. Clin. Psychol..

[11] Shadel WG (2014). Development of the PROMIS nicotine dependence item banks. Nicotine Tob. Res..

[12] Heatherton TF, Kozlowski LT, Frecker RC, Fagerström KO (1991). The Fagerström test for nicotine dependence: A revision of the Fagerström tolerance questionnaire. Br. J. Addict..

[13] Fagerström K (2012). Determinants of tobacco use and renaming the FTND to the Fagerstrom test for cigarette dependence. Nicotine Tob. Res..

[14] Fagerström K (1978). Measuring degree of physical dependence to tobacco smoking with reference to individualization of treatment. Addict. Behav..

[15] Boyle RG, Jensen J, Hatsukami DK, Severson HH (1995). Measuring dependence in smokeless tobacco users. Addict. Behav..

[16] Ebbert JO, Severson HH, Danaher BG, Schroeder DR, Glover ED (2012). A comparison of three smokeless tobacco dependence measures. Addict. Behav..

[17] Mushtaq N, Beebe LA, Vesely SK, Neas BR (2014). A multiple motive/multi-dimensional approach to measure smokeless tobacco dependence. Addict. Behav..

[18] Foulds J (2015). Development of a questionnaire for assessing dependence on electronic cigarettes among a large sample of ex-smoking E-cigarette users. Nicotine Tob. Res..

[19] Salameh P, Waked M, Aoun Z (2008). Waterpipe smoking: Construction and validation of the Lebanon Waterpipe dependence scale (LWDS-11). Nicotine Tob. Res..

[20] Agaku IT (2014). Poly-tobacco use among adults in 44 countries during 2008–2012: Evidence for an integrative and comprehensive approach in tobacco control. Drug Alcohol Depend..

[21] Petersen A, Myers MG, Tully L, Brikmanis K, Doran N (2020). Polytobacco use among young adult smokers: Prospective association with cigarette consumption. Tob. Control.

[22] Strong DR (2017). Indicators of dependence for different types of tobacco product users: Descriptive findings from wave 1 (2013–2014) of the population assessment of tobacco and health (PATH) study. Drug Alcohol Depend..

[23] Sung HY, Wang Y, Yao T, Lightwood J, Max W (2018). Polytobacco use and nicotine dependence symptoms among US adults, 2012–2014. Nicotine Tob. Res..

[24] Patrick DL (2011). Content validity–establishing and reporting the evidence in newly developed patient-reported outcomes (PRO) instruments for medical product evaluation: ISPOR PRO good research practices task force report—Part 1–eliciting concepts for a new PRO instrument. Value Health.

[25] Patrick DL (2011). Content validity–establishing and reporting the evidence in newly developed patient-reported outcomes (PRO) instruments for medical product evaluation: ISPOR PRO good research practices task force report—Part 2–assessing respondent understanding. Value Health.

[26] Morean M, Krishnan-Sarin S, O'Malley SS (2018). Comparing cigarette and e-cigarette dependence and predicting frequency of smoking and e-cigarette use in dual-users of cigarettes and e-cigarettes. Addict. Behav..

[27] Chrea C (2018). Developing fit-for-purpose self-report instruments for assessing consumer responses to tobacco and nicotine products: The ABOUT? Toolbox initiative. F1000Res.

[28] Saha TD (2010). Dimensionality of DSM-IV nicotine dependence in a national sample: An item response theory application. Drug Alcohol Depend..

[29] Kandel DB, Hu MC, Yamaguchi K (2009). Sequencing of DSM-IV criteria of nicotine dependence. Addiction.

[30] DiFranza J (2010). A systematic review of the diagnostic and statistical manual diagnostic criteria for nicotine dependence. Addict. Behav..

[31] Baker TB, Breslau N, Covey L, Shiffman S (2012). DSM criteria for tobacco use disorder and tobacco withdrawal: A critique and proposed revisions for DSM-5. Addiction.

[32] DiFranza JR (2016). Can tobacco dependence provide insights into other drug addictions?. BMC Psychiatry.

[33] Breteler MH, Hilberink SR, Zeeman G, Lammers SM (2004). Compulsive smoking: The development of a Rasch homogeneous scale of nicotine dependence. Addict. Behav..

[34] Hudmon KS (2003). A multidimensional model for characterizing tobacco dependence. Nicotine Tob. Res..

[35] DiFranza JR, Wellman RJ, Ursprung WW, Sabiston C (2009). The autonomy over smoking scale. Psychol. Addict. Behav..

[36] Richardson CG (2007). Validation of the dimensions of tobacco dependence scale for adolescents. Addict. Behav..

[37] Glover ED (2005). Developmental history of the Glover-Nilsson smoking behavioral questionnaire. Am. J. Health Behav..

[38] Yoshii C (2006). Innovative questionnaire examining psychological nicotine dependence, "the Kano test for social nicotine dependence (KTSND)". J UOEH.

[39] Hughes JR, Hatsukami D (1986). Signs and symptoms of tobacco withdrawal. Arch. Gen. Psychiatry.

[40] Prokhorov AV, Pallonen UE, Fava JL, Ding L, Niaura R (1996). Measuring nicotine dependence among high-risk adolescent smokers. Addict. Behav..

[41] O'Loughlin J (2002). Assessment of nicotine dependence symptoms in adolescents: A comparison of five indicators. Tob. Control.

[42] Horn D, Waingrow S (1966). Some dimensions of a model for smoking behavior change. Am. J. Public Health Nations Health.

[43] Davis LJ (1994). Self-administered nicotine-dependence scale (SANDS): Item selection, reliability estimation, and initial validation. J. Clin. Psychol..

[44] Heishman SJ, Singleton EG, Moolchan ET (2003). Tobacco craving questionnaire: Reliability and validity of a new multifactorial instrument. Nicotine Tob. Res..

[45] Kawakami N, Takatsuka N, Inaba S, Shimizu H (1999). Development of a screening questionnaire for tobacco/nicotine dependence according to ICD-10, DSM-III-R, and DSM-IV. Addict. Behav..

[46] Smith SS (2010). Development of the brief Wisconsin inventory of smoking dependence motives. Nicotine Tob. Res..

[47] Salameh P (2013). The young adults' cigarette dependence (YACD) score: An improved tool for cigarette dependence assessment in university students. Addict. Behav..

[48] Ikard FF, Green DE, Horn D (1969). A scale to differentiate between types of smoking as related to the management of affect. Int. J. Addict..

[49] Tønnesen P (1988). Dose and nicotine dependence as determinants of nicotine gum efficacy. Prog. Clin. Biol. Res..

[50] Heishman SJ, Singleton EG, Pickworth WB (2008). Reliability and validity of a short form of the tobacco craving questionnaire. Nicotine Tob. Res..

[51] Akers, L., Severson, H. H., Yovanoff, P. & Boles, S. M. *Oregon Research Institute Tobacco Workgroup Technical Report: Using Item Response Theory to Develop Models of Smokeless Tobacco Dependence* (Oregon Research Institute, 2011).

[52] Russell MAH, Peto J, Patel UA (1974). The classification of smoking by factorial structure of motives. R. Stat. Soc. J. Ser. A Gen..

[53] Hyland A (2017). Design and methods of the population assessment of tobacco and health (PATH) study. Tob. Control.

[54] WHO Assist Working Group (2002). The alcohol, smoking and substance involvement screening test (ASSIST): Development, reliability and feasibility. Addiction.

[55] Grant BF (2003). The alcohol use disorder and associated disabilities interview schedule-IV (AUDADIS-IV): Reliability of alcohol consumption, tobacco use, family history of depression and psychiatric diagnostic modules in a general population sample. Drug Alcohol Depend..

[56] Breslau N, Johnson EO, Hiripi E, Kessler R (2001). Nicotine dependence in the United States: Prevalence, trends, and smoking persistence. Arch. Gen. Psychiatry.

[57] Dobbs PD, Hodges EJ, Dunlap CM, Cheney MK (2020). Addiction vs. dependence: A mixed methods analysis of young adult JUUL users. Addict. Behav..

[58] Warm TA (1989). Weighted likelihood estimation of ability in item response theory. Psychometrika.

[59] Andrich D (1988). A general form of Rasch's extended logistic model for partial credit scoring. Appl. Meas. Educ..

[60] Andrich D (1978). A rating formulation for ordered response categories. Psychometrika.

[61] Mapi Research Trust. *ABOUT™—Dependence*. <https://eprovide.mapi-trust.org/instruments/about-dependence> (2023).

[62] Salzberger T (2021). Addressing traceability of self-reported dependence measurement through the use of crosswalks. Measurement.

[63] Food and Drug Administration (FDA). *Guidance for Industry—Patient-Reported Outcome Measures: Use in Medical Product Development to Support Labelling Claims* (Silver Spring, 2009).

[64] United States Census Bureau. *QuickFacts United States*. <https://www.census.gov/quickfacts/fact/table/US/PST045223> (2024).

[65] Strong DR (2020). Predictive validity of the adult tobacco dependence index: Findings from waves 1 and 2 of the population assessment of tobacco and health (PATH) study. Drug Alcohol Depend..

[66] World Health Organization (WHO). *Guidelines for Controlling and Monitoring the Tobacco Epidemic* (World Health Organization, 1998).

[67] Center for Disease Control and Prevention (CDC). *Adult Tobacco Use Information* <https://www.cdc.gov/nchs/nhis/tobacco/tobacco_glossary.htm> (2017).

[68] Fonteyn ME, Kuipers B, Grobe SJ (1993). A description of think aloud method and protocol analysis. Qual. Health Res..

[69] Willis GB (2004). Cognitive interviewing a tool for improving questionnaire design.

[70] Hobart JC, Cano SJ, Warner TT, Thompson AJ (2012). What sample sizes for reliability and validity studies in neurology?. J. Neurol..

[71] Andrich D, Marais I (2019). A Course in Rasch Measurement Theory: Measuring in the Educational, Social and Health Sciences.

[72] Lord FM, Novick MR, Birnbaum A (1968). Statistical Theories of Mental Test Scores.

[73] Rasch Unidimensional Measurement Models. *Computer software* (RUMM Laboratory Perth, 2012).

[74] IBM SPSS Statistics for Windows Version 21.0. (IBM Corp. 2012).

