# Supplementary material for: Development and initial validation of a new self-report measure to assess perceived dependence on tobacco and nicotine products
Source: Sci Rep. 2024 May 2;14:10098. doi: 10.1038/s41598-024-60790-4 (PMC11066063; doi:10.1038/s41598-024-60790-4)
Supplement: Supplementary file 1 — Supplementary Tables. [file 41598_2024_60790_MOESM1_ESM.docx]

# Supplementary Files

**Table S1. Characteristics of existing measures of perceived dependence on tobacco and nicotine products**

|  | **Name of measure** | **Year** | **Domains** | **Items** | **Applicability** | **Item**  **Generation*** | **Item**  **Reduction*** | **Psychometric analyses*** |
| --- | --- | --- | --- | --- | --- | --- | --- | --- |
|  | Reason for Smoking Scale ^1,2^ | 1966 | 6 | 23 | cigarette | LR | FA | ICR, TTR |
|  | Modified Horn-Russell Tobacco Scale^3,4^ | 1988 | 1 | 9 | cigarette | LR | FA |  |
|  | Minnesota Nicotine Withdrawal Scale^5^ | 1986 | n/a | 7 | cigarette | LR |  | TRR |
|  | Fagerström Test for Nicotine Dependence^6^ | 1991 | 1 | 6 | cigarette |  | EO | ICR, TRR, CV, KGV |
|  | Self-administered nicotine dependence scale^7^ | 1994 | 2 | 32 | cigarette | EO | FA |  |
|  | modified Fagerström Tolerance Questionnaire^8^ | 1996 | 1 | 7 | cigarette |  | FA | ICR, TRR, CV, KGV |
|  | Tobacco Dependence Screener^9^ | 1999 | 1 | 10 | cigarette | LR |  | ICR, CV, KGV |
|  | O’Loughlin scale^10^ | 2002 | 5 | 56 | cigarette | LR |  | ICR, TRR |
|  | Hooked on Nicotine Checklist^11^ | 2002 | 1 | 10 | cigarette | LR |  | ICR, TRR, CV |
|  | Cigarette Dependence Scale^12^ | 2003 | 1 | 12/5 | cigarette | LR, SI | FA | ICR, TRR, KGV |
|  | Tobacco Craving Questionnaire (long form / short form)^13,14^ | 2003 | 6/4 | 47/12 | cigarette | LR, SI |  | ICR, TRR, CV |
|  | Wisconsin Inventory of Smoking Dependence Motives (long form / short form)^15,16^ | 2004 | 13/11 | 68/37 | cigarette | LR. EO | EO, FA | ICR, CV |
|  | Nicotine Dependence Syndrome Scale^17^ | 2004 | 5 | 19 | cigarette | LR, SI, EO | EO, SI | ICR, CV |
|  | Dimensions of Tobacco Dependence Scale^18^ | 2005 | 4 | 54/35 | cigarette | LR, SI | FA | ICR, CV |
|  | Glover-Nilsson Smoking Behavioral Questionnaire^19^ | 2005 | 1 | 11 | cigarette | EO | EO, FA | ICR, TRR |
|  | Kano Test for Social Nicotine Dependence^20^ | 2006 | 2 | 10 | cigarette | EO | EO, FA | ICR, TRR, KGV, CV |
|  | Autonomy Over Smoking Scale^21^ | 2009 | 3 | 12 | cigarette | LR, SI | FA | ICR, KGV, CV |
|  | Young Adult's Cigarette Dependence ^22^ | 2013 | 4 | 16 | cigarette | LR | FA | ICR |
|  | PROMIS - Nicotine Dependence Scale (long form/short form)^23^ | 2014 | 1 | 32/8 or 4 | cigarette | LR, EO, SI | SI, EO, FA, IRT | ICR, TRR, CV, KGV |
|  | Penn State Electronic Cigarette Dependence Index^24^ | 2015 | 2 | 10 | e-cigarette | LR | SI |  |
|  | Fagerström Tolerance Questionnaire for Smokeless Tobacco ^25^ | 1995 | 1 | 9 or 10 | smokeless | EO |  | ICR, CV |
|  | Glover-Nilsson Smokeless Tobacco Behavioral Questionnaire^26^ | 2012 | 1 | 11 | smokeless |  |  | ICR, CV |
|  | Severson Smokeless Tobacco Dependency Scale^26,27^ | 2011 | 1 | 7+1 | smokeless | LR | IRT | ICR, IRT |
|  | Oklahoma Scale for Smokeless Tobacco Dependence^28^ | 2014 | 7 | 23 | smokeless | LR | FA | ICR, CV |
|  | Lebanon Waterpipe Dependence Scale^29^ | 2008 | 4 | 11 | waterpipe | LR | FA | ICR, TRR, KGVCV |
|  | Nicotine Section from the PATH Survey / Tobacco Dependence index^30,31^ | 2016/ 2017 | 1 | 24 / 16 | any TNP | LR | FA, IRT | IRT, CV, KGV |
|  | Alcohol, Smoking, and Substance Involvement Screening Test^32^ | 2002 | n/a | 7 | any TNP |  | SI | TRR, CV |
|  | Alcohol Use Disorder and Associated Disability Interview Schedule—DSM-IV^33^ | 2003 | n/a | 22 | any TNP |  |  | ICR, TRR, CV |

*****Based on information extracted from bibliographic references related to the measure development

**Abbreviations**: CV=concurrent/convergent validity; EO=expert opinion; FA=factor analysis; ICR=internal consistency reliability; IRT=item response theory; KGV=known group validity; LR=literature review; n/a=not applicable; SI=subject input; TNP: Tobacco and/or nicotine product; TRR=test-retest reliability; WHO = World Health Organization.

**Table S2. Concepts and items from the preliminary conceptual framework and qualitative research**

| **Preliminary conceptual framework from literature review and expert opinion** | **Nine-item draft measure from literature review and expert opinion** | **Concepts elicited from qualitative study with TNP users** | **Sample quotes from qualitative study with TNP users** | **19-item draft measure following qualitative study and expert panel review** |
| --- | --- | --- | --- | --- |
| Urgency to use upon waking up | …how soon after you woke up did you use your first product? | Frequency of use. | *“As soon as I wake up, it’s pretty much the first thing I reach for.”* (Male, 35-44 years, Cigar) | …how soon after you woke up did you use your first product? |
|  | …how long before going to bed did you use your last product? |  | *“It’s the last thing I do. It’s after the TV’s off, I’ve still got a couple more lights, then I put it out and I turn over.”* (Male, 45+ years, Cigarette) | …how long before going to sleep did you use your last product? |
|  | … proportion of the available time in your day did you spend using your products? |  | *“What’s this day? It could mean morning? Morning, day and night, or is it just daytime? What portion of the available time in your day… I got you. There isn’t a portion, I just smoke when I want to. … What portion? I would say the only time I couldn’t is when I’m driving and somebody is in the backseat, I wouldn’t smoke. Or if I’m with friends and they don’t like it, I’ll go outside. But there isn’t really any portion in the day where I have to think about it. In here, today, I can’t. I stay at home a lot, so I don’t have to church myself. I smoke everywhere. Portion of the day…*  [Interviewer] How would you answer that?  *I’d have to put the majority – 95% of the day I smoke.”*  (Male, 45+ years, Cigarette) | ITEM REMOVED as participants had difficulty estimating the amount of available time that they used their product. |
| Compulsion to use |  | Have to have it.  Cravings/curbs craving.  Desire to use.  Urgency to use. | *“It’s the cravings, they’re hell. Once I get that craving, then I’ve just got to smoke.”* (Male, 45+ years, Poly-user) | …feel that you HAD to have one? |
|  | …have a strong desire to use these products? |  | *“A strong desire is when I don't have any cigarettes, and sometimes it can be late at night and if I am still awake and I don't have any cigarettes, and I don't have vape, I will go to the gas station and buy some cigarettes. It doesn't matter what time it is.”* (Male, 35-44 years, Poly-user) | …have a strong desire to use your product(s)?  …hard to control the need or urge to use your product(s)? |
| Difficulty to cease using | …how difficult…to completely quit your product? | Difficulty to quit. | *“…it’s just unbelievable how hard it is to stop. You can try and it comes back to the stress level again, and it just calms you down and you are back at it again.”* (Female, 18-34 years, Poly-user) | …how difficult…to completely quit your product? |
|  |  | Difficulty to cut down | *“Well I tried to go to two cigarettes a day, and I tried to make it back to one, when I first wake up in the morning, and then that afternoon, but that didn't quite work out.” (*Female, 35-44 years, Poly-user) | …how difficult…to cut down on your product(s)? |
| Need to use to function normally | …you need your product(s) to function normally? | Reduce stress.  Increase alertness.  Time for self. | *“I need to smoke at the beginning to enable me to do my job.”* (Male, 45+ years, Poly-user)  *“You can’t function normally without having side-effects.”* (Female, 18-35 years, Poly-user) | …you need your product(s) to function normally? |
| Self-awareness of dependence | How addicted to these products do you consider yourself? | Risky beliefs.  Regret.  Part of life identity. | *“Obviously, there’s a physical dependency at some level at this point in time. But frankly, at this point in time, it’s a second nature habit.” (*Male, 35-44 years, Poly-user)  *“Character. Appearance. And I say that because, in example, my co-workers are used to seeing me with a cigar in my hand. They know that’s part of my personality, part of my character, they know I would smoke up a with a cigar.” (*Male, 35-44 years, cigar)  *“I’m not the person – I’m not the vegan. I’m not the vegetarian. I am the meat-eater. That’s who I am. I’m like give it to me. I like don’t want – give me the caffeine, don’t give me the diet, give me the real. That’s very much what I am.” (*Male, 18-34 years, Poly-user) | … how addicted to the product(s) consider yourself?  …using your product(s)…part of who you are? |
|  |  |  |  |  |
| Automaticity of using | …find yourself reaching out for these products without thinking about it? | Use more than thought. | *“I feel like I could do something and not even have to think about it: grab it out of my pocket, take a puff and put it back while still doing what I’m doing.” (*Male, 18-34 years, e-cigarette)  *“I know better with the cigars. I’m conscious of them, but I’m not conscious of my cigarettes.”* (Female, 45+ years, Poly-user) | …use more of your product(s)than you intended to?  …find yourself using your  product(s) automatically without  thinking about it)? |
| Priority of using over social responsibilities | …interrupt what you were doing so you could use these products? | Negatives related to other people. | *“…in the middle of work, sending an e-mail or whatever, sometimes I’ll stop and leave it as a draft, smoke and come back.”*  (Female, 35-44 years, Cigarette user) | …stop what you are doing to use your product(s)?  …interrupt what you are doing to use your product(s)? |
|  |  |  | *“I dip at work and I’m not necessarily supposed to be dipping at work.”* (Female, 45+ years, Poly-user) | …use your product(s) in a situation where you weren’t supposed to? |
|  |  |  | *“I would go round the side of the house so they wouldn’t know, and I smoke without them knowing. If I heard them coming, I would put it out.”* (Male, 18-34 years, Poly-user)  *“I don’t like smoking around family. I’ve done it once and I walked away, far away, and stayed away for a while then came thirty, forty minutes later and the smell and everything was gone.” (*Male, 35-44 years, Cigarette) | …sneak off to use your product?  … use your product(s) in secret?  …use an excuse so could use your product(s)? |
|  |  |  | *“Whenever we went to do stuff together or whatever, I avoided going because I know they don’t smoke.”* (Female, 18-34 years, Poly-user)  *“I have avoided going to the center just because of that once. I’ve been back before, but my friends are like why don’t you want to come and the first thing I said was if I wanted to have a smoke, I wouldn’t have the ability to.”* (Male, 35-44 years, Cigarette) | …avoid an activity because you couldn’t use your product(s)? |

**Table S3. Definitions and acceptability criteria of outcomes of Rasch Measurement Theory (RMT) analyses**

| **Property** | **Definitions and Acceptability Criteria** |
| --- | --- |
| Acceptability | Acceptability means that respondents are willing to answer the contents of the instrument while maintaining a level of attentiveness that ensures meaningful responses throughout. Since a lack of acceptability may become manifest in various ways (termination, missing responses, implausible or repetitive responses), assessment of acceptability requires a multistage procedure. Acceptability of the instrument is considered poor, if more than 20% of the participants fail to respond to more than 20% of all items. Item-level missing data should be <10%. Additionally, poor acceptability is indicated by 20% or more of the population having poor person fit statistics (person fit residual >2.5) or providing the same response throughout the scale (straight lining) suggesting inattentive or repetitive response behaviors, respectively. |
| Suitability of the Response Format of the Instrument | The extent to which the ordered response scale works as expected empirically evidenced by properly ordered threshold estimates. Thresholds mark the transition points where two adjacent response categories are equally likely. Ordered threshold estimates imply that each response category becomes the option most likely chosen by the participant in a manner that reflects the assumed order of the categories. |
| Unidimensionality of the Instrument | Unidimensionality of a set of items supposed to measure a latent variable of interest is required to justify a single estimate as a measure of that variable. Item fit is necessary but not sufficient evidence for unidimensionality. Since unidimensionality implies that item residuals (the difference between responses expected under the model and the observed responses) are uncorrelated, residual correlations are a suitable criterion of dimensionality assessment. Specifically, all residual correlations should be random and share no structure or relationship. This requirement is tested by a principal component analysis, which should not reveal eigenvalues larger than randomly expected (using eigenvalues from a parallel analysis as a benchmark). |
| Item Hierarchy | Based on the overall location of each item as the mean of its threshold estimates, the hierarchy of items characterizes the meaning of the latent variable and what represents less and what represents more of the construct. The empirical order of items is scrutinized in terms of its conceptual meaningfulness and interpretation. |
| Targeting | Targeting is the extent to which the items in a scale match the participant population in terms of the variable measured. Perfect targeting implies that all participant measures are within the range of item threshold estimates. Good targeting allows for precise measurement (small standard errors) with few extreme scores and differentiation between participants. Thus, targeting is evaluated in terms of the number of extreme scores (should be less than 10% at the bottom and at the top end, respectively) and the matching of item threshold locations and participant locations. |
| Person Separation | Person-separation is the extent to which a set of items separates participants and allows for precise measurement. It is assessed by means of the person-separation-index (PSI), which is the Rasch Measurement Theory equivalent of traditional reliability being defined by the ratio of the estimated true variance over the total observed variance of the participant measures. The interpretation of the size of the PSI corresponds to the interpretation of traditional reliability in CTT. Thus, the PSI should at least reach 0.7. |
| Item Discrimination | Item discrimination assesses the extent to which each item matches the discrimination of other items as required by Rasch measurement theory. An approximately normally distributed fit residual statistic between -2.5 and +2.5, derived from item residuals, indicates proper item discrimination. Values >2.5 point at under-discrimination as an indication of poor item quality or multidimensionality. Values <-2.5 are indicative of over-discrimination, typically as a result of local dependence or multidimensionality. In large samples, fit residual statistics tend to be inflated and should be interpreted with caution and in conjunction with other evidence of local dependence and multidimensionality. |
| Item Fit | Item fit is concerned with whether observed responses match expected responses under the Rasch model. Comparing expected and observed responses in class intervals (groups of homogeneous participants) yields an approximately chi-square distributed fit statistic. Its p-value shows the probability of the response data given perfect fit. A cut-off value for a type-one error of 0.01 is generally deemed appropriate with values between 0.001 and 0.01 still being acceptable if no other indicators of fit suggest model violations. As in large samples (n>500) acceptable items may show significant misfit, fit should be assessed for a reasonable sample size. |
| Person Fit | Person fit assesses the extent to which observed individual response patterns of the participants match the response patterns expected under the model. Person fit is investigated as part of the acceptability of the instrument. |
| Local Independence | Local independence implies that item responses are unrelated once their common cause, the variable to be measured, has been accounted for. Therefore, local independence means that item residual correlations should be near zero. Since the total score is given, the mean of all residual correlations is smaller than 0. Residual correlations larger than +0.3 above the mean of all correlations are considered strong evidence of local dependence, while items for which the residual correlations are between +0.2 and +0.3 above the mean are scrutinized for possible redundancy. |
| Item Invariance | Invariance, or the mutual independence of item and person properties, is a fundamental requirement of generalizable measurement. Being a defining property of the Rasch model, item invariance means that item parameter estimates do not depend on participant characteristics. Invariance can be tested empirically by comparing mean residual responses of different participants groups (for example different TNP users) in class intervals of homogeneous participants using a two-way analysis of variance (with class interval and the participant grouping variable as main effects). A significant main effect of the participant grouping variable, or a significant interaction effect implies differential item functioning (DIF). While small DIF might not result in meaningful differences, DIF generally requires corrective action as it is a threat to comparability of measurements. Splitting the item based on the participant group allows for estimating group-specific item parameters as a remedy. |

**Table S4.** **Item Reduction based on Rasch Measurement Theory (RMT) analyses**

| **Item** | **Statement (brief)** | **Behavioral Impact**  (11^a^ − 2^b^) | **Signs and Symptoms**  (8) | **Extent of Use**  (2^c^) | **Decision for reduction** |
| --- | --- | --- | --- | --- | --- |
| TD01 | After waking up | (x) |  | **x** | RETAINED in EXTENT OF USE domain (under-discriminating in the context of all Behavioral Impact items) |
| TD02 | Before sleep | (x) |  | **x** | RETAINED in EXTENT OF USE domain (under-discriminating in the context of all Behavioral Impact items) |
| **TD03** | **Function normally** |  | **x** |  | RETAINED |
| TD04 | Difficulty to cut down |  | x |  | Eliminated due to redundancy with TD06 |
| TD05 | Perception of whether addicted |  | x |  | Eliminated due to over-discrimination, misfit and redundancy with item TD06 |
| **TD06** | **Difficulty to quit** |  | **x** |  | RETAINED |
| TD07 | Part of who you are |  | x |  | Eliminated due to misfit |
| **TD08** | **Strong desire** |  | **x** |  | RETAINED |
| **TD09** | **Use more than intended** | **x** |  |  | RETAINED |
| **TD10** | **Had to have one** |  | **x** |  | RETAINED |
| **TD11** | **Use in situations not supposed to** | **x** |  |  | RETAINED |
| TD12 | Excuse | x |  |  | Eliminated due to redundancy with TD16 and misfit |
| **TD13** | **Urge hard to control** |  | **x** |  | RETAINED |
| TD14 | Use in secret | x |  |  | Eliminated due to redundancy with TD16 |
| TD15 | Interrupt what you are doing | x |  |  | Eliminated due to redundancy with TD18 and misfit |
| **TD16** | **Sneak off to use** | **x** |  |  | RETAINED |
| **TD17** | **Avoid activity can't use** | **x** |  |  | RETAINED |
| **TD18** | **Stop what doing to use** | **x** |  |  | RETAINED |
| TD19 | Using automatically | x |  |  | Eliminated due to redundancy with TD09 and misfit |

^a^ First grouping (11 items)

^b^ Second grouping (9 items)

^c^ Second grouping (2 items)

TD refers to measure’s item number. Items in **bold** indicate the final retained items for the Behavioral Impact (5 items), Signs and Symptoms (5 items) and Extent of Use (2 items) domains.

**Table S5. Definitions and acceptability criteria of outcomes of Classical Test Theory (CTT) analyses**

| **Property** | **Definitions and Acceptability Criteria** |
| --- | --- |
| Scaling Assumptions | Scaling assumptions refer to the extent to which it is legitimate to sum a set of item scores, without weighting or standardization, to produce a single total score. An unweighted sum score is justified when all items measure the same underlying unidimensional variable, have similar item means and variances. All items should show a corrected item-total correlation (ITC) ≥ 0.30, which should also be of similar magnitude. |
| Unidimensionality | Unidimensionality means that there is one underlying latent variable accounting for the observed item scores, assessed empirically by exploratory principal axis factor analysis. Unidimensionality requires a large and dominant eigenvalue of the first factor investigated by a visual inspection of the scree plot showing eigenvalues in decreasing order. In addition, only the first eigenvalue should exceed the expected eigenvalue from a parallel analysis under the assumption of entirely uncorrelated data. |
| Targeting | At the level of the sum score, proper targeting requires that scale scores should span the entire range and are approximately symmetrically distributed. Thus, floor and ceiling effects (proportions of the sample at the minimum and maximum scale score, respectively) should be low (<15%). Skewness statistics should range from –1 to +1. There are no specific criteria for item level targeting. Therefore, scale-level criteria of floor and ceiling effects, and skewness, can be applied. |
| Internal Consistency Reliability | Reliability is defined as the ratio of true variance in the participant scores over total variance being the sum of true variance and error variance. It refers to the extent to which scale scores reflect random error. Reliability is estimated as internal consistency reliability by means of coefficient alpha (Cronbach’s alpha), which is based on the intercorrelations of items. A reliability of 0.7 is frequently suggested as a lower boundary for acceptable reliability for group comparisons. However, this criterion only applies at an early stage and does not allow for precise measurement at the individual level. For important decisions to be based on instrument scores at the individual level, Nunnally proposes a minimum alpha of 0.90 or even 0.95. |
| Stability over time | Stability (test-retest reliability) over time is assessed by the correlation of measures estimated at different points in time. |

## **References**

1 Horn, D. & Waingrow, S. Some dimensions of a model for smoking behavior change. *Am J Public Health Nations Health* **56**, Suppl 56:21-56, doi:10.2105/ajph.56.12_suppl.21 (1966).

2 Ikard, F. F., Green, D. E. & Horn, D. A Scale to Differentiate between Types of Smoking as Related to the Management of Affect. *International Journal of the Addictions* **4**, 649-659, doi:10.3109/10826086909062040 (1969).

3 Russell, M. A. H., Peto, J. & Patel, U. A. The Classification of Smoking by Factorial Structure of Motives. *Royal Statistical Society. Journal. Series A: General* **137**, 313-346, doi:10.2307/2344953 (1974).

4 Tønnesen, P. Dose and nicotine dependence as determinants of nicotine gum efficacy. *Prog Clin Biol Res* **261**, 129-144 (1988).

5 Hughes, J. R. & Hatsukami, D. Signs and symptoms of tobacco withdrawal. *Arch Gen Psychiatry* **43**, 289-294, doi:10.1001/archpsyc.1986.01800030107013 (1986).

6 Heatherton, T. F., Kozlowski, L. T., Frecker, R. C. & Fagerström, K. O. The Fagerström Test for Nicotine Dependence: a revision of the Fagerström Tolerance Questionnaire. *Br J Addict* **86**, 1119-1127, doi:10.1111/j.1360-0443.1991.tb01879.x (1991).

7 Davis, L. J., Jr. *et al.* Self-administered Nicotine-Dependence Scale (SANDS): item selection, reliability estimation, and initial validation. *J Clin Psychol* **50**, 918-930, doi:10.1002/1097-4679(199411)50:6<918::aid-jclp2270500617>3.0.co;2-6 (1994).

8 Prokhorov, A. V., Pallonen, U. E., Fava, J. L., Ding, L. & Niaura, R. Measuring nicotine dependence among high-risk adolescent smokers. *Addict Behav* **21**, 117-127, doi:10.1016/0306-4603(96)00048-2 (1996).

9 Kawakami, N., Takatsuka, N., Inaba, S. & Shimizu, H. Development of a screening questionnaire for tobacco/nicotine dependence according to ICD-10, DSM-III-R, and DSM-IV. *Addict Behav* **24**, 155-166, doi:10.1016/s0306-4603(98)00127-0 (1999).

10 O'Loughlin, J. *et al.* Assessment of nicotine dependence symptoms in adolescents: a comparison of five indicators. *Tob Control* **11**, 354-360, doi:10.1136/tc.11.4.354 (2002).

11 Wellman, R. J. *et al.* Measuring adults' loss of autonomy over nicotine use: the Hooked on Nicotine Checklist. *Nicotine Tob Res* **7**, 157-161, doi:10.1080/14622200412331328394 (2005).

12 Etter, J. F., Le Houezec, J. & Perneger, T. V. A self-administered questionnaire to measure dependence on cigarettes: the cigarette dependence scale. *Neuropsychopharmacology* **28**, 359-370, doi:10.1038/sj.npp.1300030 (2003).

13 Heishman, S. J., Singleton, E. G. & Moolchan, E. T. Tobacco Craving Questionnaire: reliability and validity of a new multifactorial instrument. *Nicotine Tob Res* **5**, 645-654, doi:10.1080/1462220031000158681 (2003).

14 Heishman, S. J., Singleton, E. G. & Pickworth, W. B. Reliability and validity of a Short Form of the Tobacco Craving Questionnaire. *Nicotine Tob Res* **10**, 643-651, doi:10.1080/14622200801908174 (2008).

15 Piper, M. E. *et al.* A multiple motives approach to tobacco dependence: the Wisconsin Inventory of Smoking Dependence Motives (WISDM-68). *J Consult Clin Psychol* **72**, 139-154, doi:10.1037/0022-006x.72.2.139 (2004).

16 Smith, S. S. *et al.* Development of the Brief Wisconsin Inventory of Smoking Dependence Motives. *Nicotine Tob Res* **12**, 489-499, doi:10.1093/ntr/ntq032 (2010).

17 Shiffman, S., Waters, A. & Hickcox, M. The nicotine dependence syndrome scale: a multidimensional measure of nicotine dependence. *Nicotine Tob Res* **6**, 327-348, doi:10.1080/1462220042000202481 (2004).

18 Richardson, C. G. *et al.* Validation of the Dimensions of Tobacco Dependence Scale for adolescents. *Addict Behav* **32**, 1498-1504, doi:<https://doi.org/10.1016/j.addbeh.2006.11.002> (2007).

19 Glover, E. D. *et al.* Developmental history of the Glover-Nilsson smoking behavioral questionnaire. *Am J Health Behav* **29**, 443-455, doi:10.5555/ajhb.2005.29.5.443 (2005).

20 Yoshii, C. *et al.* Innovative questionnaire examining psychological nicotine dependence, "The Kano Test for Social Nicotine Dependence (KTSND)". *J UOEH* **28**, 45-55, doi:10.7888/juoeh.28.45 (2006).

21 DiFranza, J. R., Wellman, R. J., Ursprung, W. W. & Sabiston, C. The Autonomy Over Smoking Scale. *Psychol Addict Behav* **23**, 656-665, doi:10.1037/a0017439 (2009).

22 Salameh, P. *et al.* The Young Adults' Cigarette Dependence (YACD) score: an improved tool for cigarette dependence assessment in university students. *Addict Behav* **38**, 2174-2179, doi:10.1016/j.addbeh.2013.01.009 (2013).

23 Shadel, W. G. *et al.* Development of the PROMIS nicotine dependence item banks. *Nicotine Tob Res* **16 Suppl 3**, S190-201, doi:10.1093/ntr/ntu032 (2014).

24 Foulds, J. *et al.* Development of a questionnaire for assessing dependence on electronic cigarettes among a large sample of ex-smoking E-cigarette users. *Nicotine Tob Res* **17**, 186-192, doi:10.1093/ntr/ntu204 (2015).

25 Boyle, R. G., Jensen, J., Hatsukami, D. K. & Severson, H. H. Measuring dependence in smokeless tobacco users. *Addict Behav* **20**, 443-450, doi:10.1016/0306-4603(95)00013-3 (1995).

26 Ebbert, J. O., Severson, H. H., Danaher, B. G., Schroeder, D. R. & Glover, E. D. A comparison of three smokeless tobacco dependence measures. *Addict Behav* **37**, 1271-1277, doi:10.1016/j.addbeh.2012.06.011 (2012).

27 Akers, L., Severson, H. H., Yovanoff, P. & Boles, S. M. Oregon Research Institute Tobacco Workgroup Technical Report: Using Item Response Theory to Develop Models of Smokeless Tobacco Dependence., (Oregon Research Institute, Eugene, Oregon, 2011).

28 Mushtaq, N., Beebe, L. A., Vesely, S. K. & Neas, B. R. A multiple motive/multi-dimensional approach to measure smokeless tobacco dependence. *Addict Behav* **39**, 622-629, doi:10.1016/j.addbeh.2013.11.016 (2014).

29 Salameh, P., Waked, M. & Aoun, Z. Waterpipe smoking: construction and validation of the Lebanon Waterpipe Dependence Scale (LWDS-11). *Nicotine Tob Res* **10**, 149-158, doi:10.1080/14622200701767753 (2008).

30 Hyland, A. *et al.* Design and methods of the Population Assessment of Tobacco and Health (PATH) Study. *Tob Control* **26**, 371-378, doi:10.1136/tobaccocontrol-2016-052934 (2017).

31 Strong, D. R. *et al.* Indicators of dependence for different types of tobacco product users: Descriptive findings from Wave 1 (2013-2014) of the Population Assessment of Tobacco and Health (PATH) study. *Drug Alcohol Depend* **178**, 257-266, doi:10.1016/j.drugalcdep.2017.05.010 (2017).

32 WHO ASSIST Working Group. The Alcohol, Smoking and Substance Involvement Screening Test (ASSIST): development, reliability and feasibility. *Addiction* **97**, 1183-1194, doi:10.1046/j.1360-0443.2002.00185.x (2002).

33 Grant, B. F. *et al.* The Alcohol Use Disorder and Associated Disabilities Interview Schedule-IV (AUDADIS-IV): reliability of alcohol consumption, tobacco use, family history of depression and psychiatric diagnostic modules in a general population sample. *Drug Alcohol Depend* **71**, 7-16, doi:10.1016/s0376-8716(03)00070-x (2003).
